# Urea immunoliposome inhibits human vascular endothelial cell proliferation for hemangioma treatment

**DOI:** 10.1186/1477-7819-11-300

**Published:** 2013-11-23

**Authors:** Zhiliang Wang, Jie Li, Xin Xu, Xianglong Duan, Gang Cao

**Affiliations:** 1 Department of General Surgery, The Second Affiliated Hospital of Medical College, Xi’an Jiaotong University, Xi’an 710004, China

**Keywords:** Anti-VEGFR, HVECs, Hemangioma, Immunoliposome, Targeted therapy, Urea

## Abstract

**Background:**

Urea injection has been used in hemangioma treatment as sclerotherapy. It shrinks vascular endothelial cells and induces degeneration, necrosis, and fibrosis. However, this treatment still has disadvantages, such as lacking targeting and difficulty in controlling the urea dosage. Thus, we designed a urea immunoliposome to improve the efficiency of treatment.

**Methods:**

The urea liposome was prepared by reverse phase evaporation. Furthermore, the urea immunoliposome was generated by coupling the urea liposome with a vascular endothelial growth factor receptor (VEGFR) monoclonal antibody using the glutaraldehyde cross-linking method. The influence of the urea immunoliposome on cultured human hemangioma vascular endothelial cells was observed preliminarily.

**Results:**

Urea immunoliposomes showed typical liposome morphology under a transmission electron microscope, with an encapsulation percentage of 54.4% and a coupling rate of 36.84% for anti-VEGFR. Treatment with the urea immunoliposome significantly inhibited the proliferation of hemangioma vascular endothelial cells (HVECs) in a time- and dose-dependent manner.

**Conclusions:**

The urea immunoliposome that we developed distinctly and persistently inhibited the proliferation of HVECs and is expected to be used in clinical hemangioma treatment.

## Background

Hemangioma is the most common benign tumor of infancy and is characterized by vascular endothelial cell proliferation. Because its predilection sites are always on the body surface, especially in the maxillofacial region, hemangioma seriously affects patients’ appearance and physiological function, and even induces mental stress and psychological disorders [1,2]. Urea injection has long been used in the treatment of hemangioma in our hospital as a type of sclerotherapy, particularly in refractory and recurrent cases [3-5]. Urea can shrink hemangioma endothelial cells to induce degeneration, necrosis, and fibrosis. This treatment has shown obvious therapeutic effects, even cures. However, it still presents several disadvantages: urea injection is not targeted, and its dosage is difficult to control: too high a dose would induce local necrosis, ulceration, and infection, ultimately inducing scars and developmental disorders; too low a dose would not induce an obvious effect and would prolong the treatment cycle. Inappropriately high local urea doses have also induced other complications [3].

Vascular endothelial growth factor (VEGF) and its receptor (VEGFR) have been reported to be crucial in vasculogenesis and angiogenesis. They are closely related with the pathology of hemangioma [6-8]. VEGF is secreted from vascular endothelial cells (VECs), as well as some non-endothelial cells. Levels of VEGF are increased in proliferative hemangioma specimens and decreased in paracmastic specimens [2]. As a specific mitogen of vascular endothelial cells, VEGF binds to VEGFR on VECs and promotes endothelial cell mitosis, proliferation, and migration, and the formation of new vessels in cooperation with non-endothelial cells.

In this study, we designed a urea immunoliposome that consisted of encapsulated urea with liposomes [9,10] coupled with an anti-VEGFR antibody. The liposome is designed to protect the urea, delay its degradation, and help its incorporation into human hemangioma vascular endothelial cells (HVECs). The coupled VEGFR monoclonal antibody ensures the targeting of urea to VECs, in order to improve the efficacy of urea and reduce the complications of urea injection. The influence of our urea immunoliposome on cultured human HVECs was observed preliminarily.

## Methods

### Urea liposome preparation

The urea liposome was prepared by reverse phase evaporation. Soybean phospholipid (Taiwei, Shanghai, China) and cholesterol (Yansheng, Shanghai, China) were first mixed at a weight ratio of 4:1 and dissolved in 5 ml of ethyl ether. Subsequently, 2.4 g of urea (the Second Affiliated Hospital of Medical College, Xi’an Jiaotong University) and 5 ml of distilled water were added. They were mixed ultrasonically for 10 min until a stable water/oil emulsion was formed. The ether was then removed by vacuum at 25°C. Five milliliters of distilled water were added, and the resultant mixture was successively filtered through 0.8 μm and 0.45 μm membranes. Finally, 10 ml of 24% urea liposome was prepared and stored at 4°C for use.

### Encapsulation percentage determination

Five hundred microliters of urea liposome were loaded on the top of a Sephadex-50 column (Pharmacia, Philadelphia, PA) and eluted with deionized water. The eluate was successively collected into ten tubes, 3 ml in each tube, at a dropping rate of 1 ml/min. Chromatography was performed at room temperature and developed by paradimethylaminobenzaldehyde for 10 min. The absorbance values of each eluate were determined using a spectrophotometer at a wavelength of 440 nm. The encapsulation percentage of the urea liposome was calculated with the formula:

$$\begin{array}{l} \text{encapsulation percentage}\;\left(\%\right)\\ \;=\frac{\text{quantity of urea encapsulated in lipsome}}{\text{quantity of total urea}}\times 100\% \end{array}$$

### Urea immunoliposome preparation

Twenty-five microliters of 25% glutaraldehyde was added to 1 ml of 24% urea liposome, and the mixture was maintained at 20°C for 10 min. The excess glutaraldehyde was removed by saline dialysis for 2 h. Subsequently, the saline was exchanged for fresh saline containing 0.5 mg of anti-VEGFR2 (R&D systems, Minneapolis, MN), and the dialysis proceeded overnight at 4°C. After sterilization by ^60^Co irradiation, 24% urea immunoliposome was prepared and stored at 4°C for use.

### Coupling rate determination

The urea immunoliposomes were separated via six separations by sucrose density gradient centrifugation. The phospholipid and protein contents in each separation were determined by the molybdenum blue and Coomassie methods, respectively. The second separation contained both lipids and proteins. Only proteins were detected in the fifth and sixth separations. Thus, the second separation, which contained both lipids and protein, harbored the immunoliposomes composed of anti-VEGFR coupled with the urea liposome. The coupling rate of the urea immunoliposome was calculated with the formula:

$$\begin{array}{l} \text{coupling rate}\;\left(\%\right)\\ \;=\frac{\text{content of anti}‒\text{VEGFR coupled with uread lipsome}}{\text{initial content of anti}‒\text{VEGFR}}\times 100\% \end{array}$$

### Morphology of urea immunoliposome was observed by transmission electron microscopy

A 24% urea immunoliposome solution was separately diluted 10 times and 100 times. One drop of each dilution was added to a Form wan copper net and stained negatively by 2% phosphotungstic acid. After drying at room temperature, the nets were examined with an electron microscope (TEM-2000EX, JEOL, Japan).

### Hemangioma vascular endothelial cell preparation and culture

The experiment was approved by the ethics committees of Xi’an Jiaotong University School of Medicine. Informed consent was obtained from all patients. Resected tissue obtained from surgery on children aged 59 days suffering from strawberry hemangioma on the abdominal surface was selected to isolate the HVECs for tissue culture. The hemangiomas were cut into small pieces, inoculated into culture flasks, and maintained in DMEM medium (GIBCO, NY) supplemented with 10% FBS (Sijiqing, Hangzhou, China) at 37°C in a humidified atmosphere of 5% CO_2_ and 95% air. After 3 days, the endothelial cells quickly migrated from the tissue pieces. One week later, some endothelial cells became confluent. After removing the tissue pieces, the endothelial cells formed a monolayer, covering the bottom of the flask after approximately 3 weeks. They were then subcultured by trypsin digestion and grew actively as the number of passages increased. Compared with primary cells, endothelial cells showed a more stretched, spindle-like morphology after several passages, but still grew in a monolayer. They were cultured *in vitro* for approximately 6 months. The HVECs we isolated and cultured were identified by HVEC factor VIII related antigen (VIII-R Ag) immunostaining and VEGFR2 flow cytometry.

For the immunostaining with VIII-R Ag, subcultured endothelial cells were inoculated on a cover glass and incubated for 10 min in 0.3% H_2_O_2_ diluted in methanol to reduce endogenous peroxidase activity. After blocking with normal goat serum, they were then incubated with anti-VIII-R Ag (1:50 dilution, ZSBIO, Beijing, China), followed by an incubation with secondary antibody conjugated with biotin (ZSBIO) and development with ABC (ZSBIO) and diaminobenzidine reagent (Boster, Wuhan, China). Digital images were obtained using a Leica Photo Microscope (Q550CW, Leica, Germany).

For VEGFR flow cytometry, subcultured endothelial cells were suspended in cold culture medium at a density of 5 × 10^6^ cells per milliliter. Forty microliters of cell suspension were mixed with anti-VEGFR2 and maintained at 4°C for 30 min. After washing, they were incubated with secondary antibody conjugated with fluorescein isothiocyanate (Boster) at 4°C for 30 min. The cells were analyzed with an automated fluorescence-activated cell counter (Elite, Beckman Coulter), with which 1,000,000 events were counted. The absolute number of cells expressing VEGFR2 per 1,000,000 events was calculated, and the percentage was derived.

### Influence of urea immunoliposome on human vascular endothelial cell morphology

The HVECs were passaged in 96-well plates at a density of 4 × 10^3^ cells per well. Twenty-four hours later, the urea immunoliposomes diluted with culture medium were added to each well at different final concentrations of 0, 0.002%, 1%, 2%, 3%, 4%, 5%, 10%, 20%, or 40%. Four wells were seeded per concentration. Changes in HVEC morphology were observed under an inverted phase contrast microscope (IMT-2, Olympus, Japan) after 24 h, 48 h, and 72 h.

### Influence of urea immunoliposome on human vascular endothelial cell proliferation

The HVECs were passaged in 96-well plates at a density of 2 × 10^3^ cells per well. Twenty-four hours later, urea immunoliposomes diluted with culture medium were added to each well at the same final concentrations, mentioned previously. After 24, 48, and 72 hours, the proliferation of HVECs was measured by the MTT method (Sigma) according to the manufacturer’s protocols. The median influence dose (ID_50_) was calculated by linear regression analysis.

The HVECs were passaged in 24-well plates at a density of 1 × 10^4^ cells per well. Twenty-four hours later, the cells were divided into five groups: three experimental groups treated with 2.6% urea, 2.6% urea liposome, or 2.6% urea immunoliposome, and two control groups, treated with the same volume of liposome or culture medium, at four wells per group. Subsequently, the number of HVECs in each group was counted every day for 8 days. A growth curve was generated and the population doubling time was calculated using the following formula:

$$\mathrm{doubling}\;\mathrm{time}=t\times \log2/\left(\log N_{t}-\log N_{0}\right)$$

where *t* represents the culture period during which the cell grows in the log phase; *N*_0_ is the cell number of cells at the start of the log phase; and *N*_*t*_ is the cell number at the end of the log phase. On the eighth day, the cell numbers of each group were used to calculate the inhibition rate:

$$\begin{array}{l} \text{inhibition rate}\;\left(\%\right)\\ =\frac{\left(\text{cell number of control group}-\text{cell number of experimental group}\right)}{\text{cell number of control group}}\times 100\% \end{array}$$

### Data analysis

Data are expressed as the mean ± standard errors of the mean with the number of individual experiments described in the figure legends. The differences between groups were analyzed using Student’s *t* test. *P* < 0.05 was considered statistically significant.

## Results

### Urea immunoliposome characteristics

**Figure 1:**
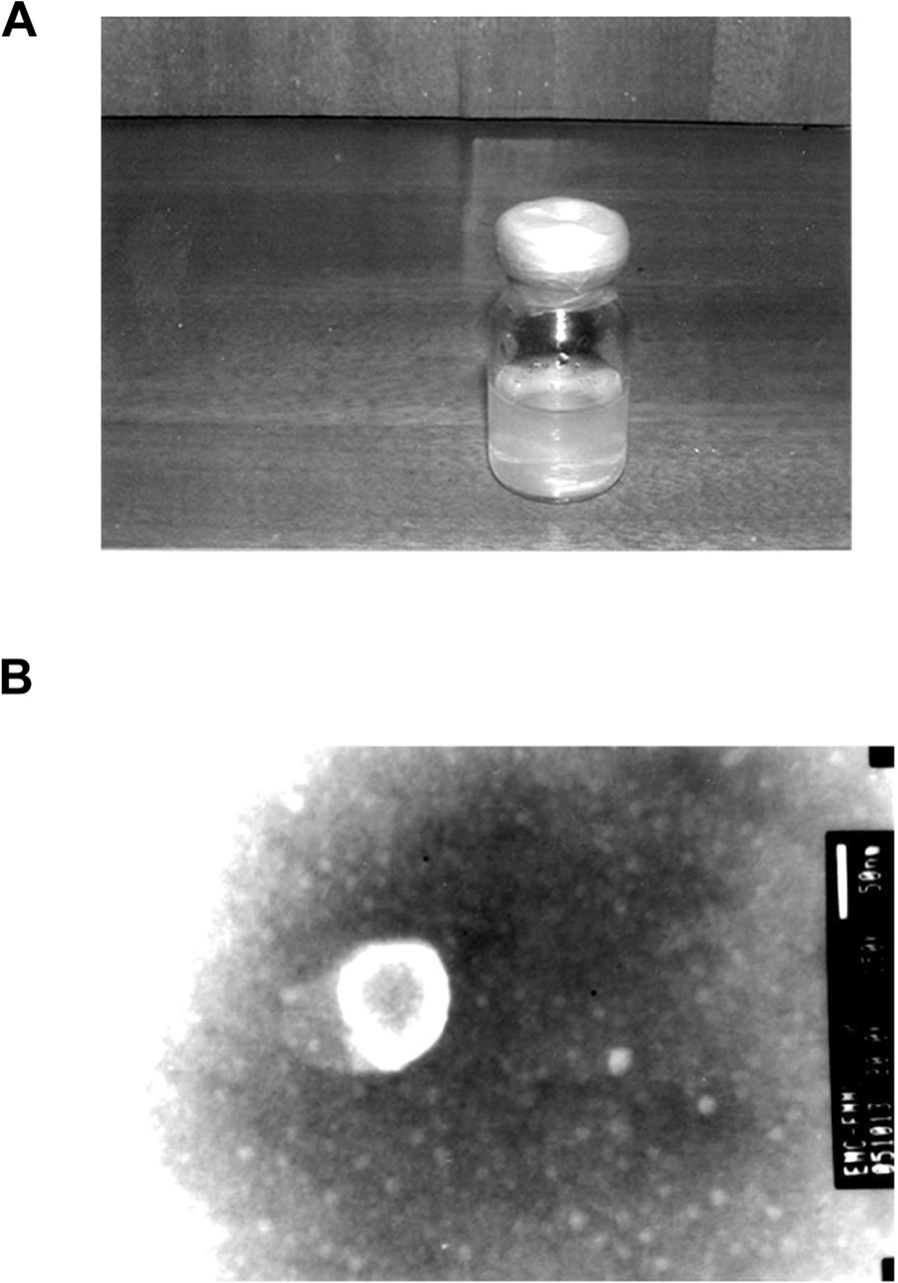
**Urea immunoliposome morphology. (A)** Both the urea liposome and urea immunoliposome generated a suspension that appeared milky white upon gross inspection. **(B)** Urea immunoliposomes diluted 100 times observed under a transmission electron microscope (×150,000).

Both the urea liposome and urea immunoliposome formed a milky white suspension on gross examination, as shown in Figure 1A, which remained stable and did not obviously change in appearance for 6 months at 4°C. The urea immunoliposomes diluted 100 times showed a typical liposome morphology under a transmission electron microscope, as shown in Figure 1B. The urea immunoliposomes were spherical or near spherical, large unilamellar liposomes with a diameter of 150 to 200 nm. A nucleolar structure and lipid-coating structure were obvious. The encapsulation percentage of urea liposomes was 54.4%, according to Sephadex-50 column elution analysis. The coupling rate of anti-VEGFR to urea immunoliposomes was 36.84%.

### Preparation and culture of hemangioma vascular endothelial cells

**Figure 2:**
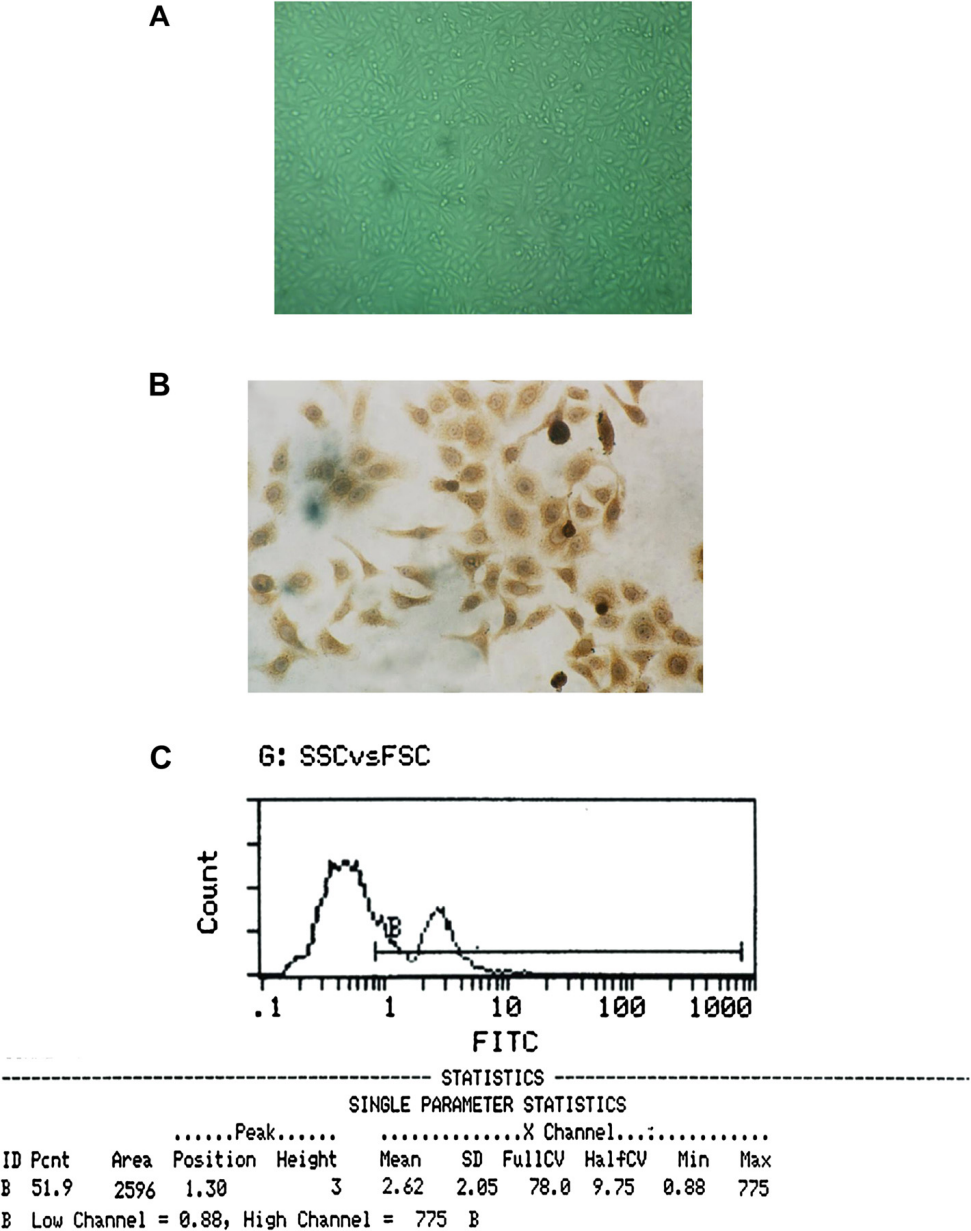
**Hemangioma vascular endothelial cell (HVEC) culture and identification. (A)** The HVECs were derived from tissues resected during the surgery of children aged 59 days who had strawberry hemangioma on the abdominal surface. The tissues were cut into small pieces, inoculated into culture flasks, and maintained in culture medium. After 3 days, the endothelial cells migrated quickly from the tissue pieces. One week later, some of the endothelial cells became confluent (×100). **(B)** The HVECs were identified with human vascular endothelial cells factor VIII related antigen (VIII-R Ag) immunostaining (×200). **(C)** The HVECs were also analyzed with VEGFR2 flow cytometry. The percentage of HVECs in cells that we isolated and subcultured was 59.1%.

The HVECs were isolated by tissue culture. Approximately 3 days after inoculation, the endothelial cells migrated quickly from the tissue pieces and showed a polygonal structure, clear boundaries, abundant cytoplasm, and central round or oval nuclei. One week later, some of the endothelial cells became confluent (Figure 2A). After removing the tissue pieces, the endothelial cells grew as a monolayer, covering the bottom of the flask approximately 3 weeks later. They were then subcultured by trypsin digestion and grew actively as the number of passages increased. Compared with primary cells, endothelial cells were more stretched and spindle-like after several passages, but still grew in a monolayer. Immunostaining with VIII-R Ag showed that the cells that we isolated and subcultured were positively stained (Figure 2B). The data obtained from the VEGFR2 flow cytometry indicated that the percentage of HVECs in cells we isolated and subcultured was 51.9% (Figure 2C), which was identical to the figures reported in previous reports [2].

### Influence of urea immunoliposome on HVEC proliferation

The HVECs were damaged by urea immunoliposome at a concentration of 40% and instantly died. The treatment time did not obviously influence the other groups. Twenty-four hours later, most of the HVECs treated with 10% and 20% urea immunoliposome were dead, and HVECs treated with 5% urea immunoliposome were beginning to die. The other treatment groups did not show signs of cell death. Forty-eight hours later, some of the HVECs treated with 1%, 2%, 3%, and 4% urea immunoliposome were dead and continued to die as the treatment time was extended. The HVECs treated with 0.002% urea immunoliposome did not show obvious changes during the entire 72 hours compared with the control. The proliferation of HVECs was inhibited by urea immunoliposomes, and the ID_50_ was obtained from the linear regression equation:

$${\mathrm{ID}}_{50}=\left(1.4024–1.368\times 50\%\right)/0.02756=2.6\%.$$

**Figure 3:**
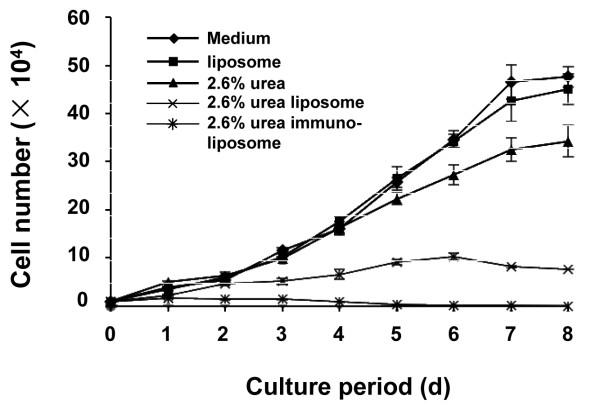
**Urea immunoliposome induced inhibition of hemangioma vascular endothelial cell (HVEC) proliferation.** HVECs were passaged in 24-well plates at a density of 1 × 10^4^ cells per well. Twenty-four hours later, the cells were divided into five groups: three experimental groups treated with 2.6% urea, 2.6% urea liposome, or 2.6% urea immunoliposome, and two control groups treated with the same volume of liposome or normal medium at four wells per group. The number of cultured HVECs was subsequently counted every day for 8 days. The data are representative of three independent experiments.

Cell proliferation was inhibited by 2.6% urea, 2.6% urea liposome, and 2.6% urea immunoliposome, as shown in Table 1 and Figure 3. The HVECs in the urea immunoliposome group were most influenced, followed by the urea liposome group, and the urea group. The two control groups, HVECs treated with culture medium or liposomes, did not show obvious differences. Compared with the control, the inhibition rate of HVEC proliferation on the 8th day was 99.91% in the urea immunoliposome group, 84.01% in the urea liposome group, and 27.94% in the urea group. The population doubling time in the medium, liposome, urea, and urea liposome groups were 43.1 h, 49.5 h, 63.7 h, and 74.4 h, respectively. The doubling time could not be obtained for the urea immunoliposome group, owing to the severely inhibited proliferation.

**Table 1:** The proliferation of HVECs was inhibited by urea, urea liposome, and urea immunoliposome treatment

**Culture period (day)**	**Cell number (*X*** **±** ***S*, ×10**^**4**^**)**				
	**Medium**	**Liposome**	**2.6% urea**	**2.6% urea liposome**	**2.6% urea immunoliposome**
0	1.00 ± 0.15	1.00 ± 0.14	1.00 ± 0.05	1.00 ± 0.28	1.00 ± 0.24
1	3.90 ± 0.19	3.62 ± 0.17	5.00 ± 0.26	2.25 ± 0.11*	1.75 ± 0.09**
2	5.43 ± 0.45	5.89 ± 0.24	6.30 ± 0.86	4.64 ± 0.32*	1.40 ± 0.11***
3	11.6 ± 0.50	10.25 ± 0.72	9.98 ± 0.28	5.16 ± 0.64**	1.40 ± 0.07***
4	16.01 ± 0.92	17.50 ± 1.12	16.19 ± 1.38	6.56 ± 1.02***	0.86 ± 0.04***
5	25.73 ± 1.07	26.5 ± 2.41	22.31 ± 1.15*	9.10 ± 0.52***	0.27 ± 0.03***
6	34.65 ± 1.73	34.25 ± 1.42	27.30 ± 2.13*	10.24 ± 0.73***	0.19 ± 0.01***
7	46.50 ± 3.57	42.60 ± 4.21	32.46 ± 2.45**	8.14 ± 0.23***	0.08 ± 0.0038***
8	47.6 ± 2.10	45.00 ± 3.20	34.30 ± 3.30**	7.61 ± 0.003***	0.04 ± 0.0021***

HVECs were treated as in Figure 3. The daily cell numbers are shown as the mean ± standard error in the mean and are representative of three independent experiments. *P < 0.05; **P < 0.01; ***P < 0.0001, compared with the control.

## Discussion

Urea injection has been used to treat hemangioma in our hospital as a type of sclerotherapy. Urea can shrink hemangioma endothelial cells to induce degeneration, necrosis, and fibrosis. However, this treatment still features disadvantages, such as a lack of targeting and difficulty in controlling the urea dosage. Thus, we designed a urea immunoliposome that consisted of urea encapsulated with a liposome that was coupled with a VEGFR monoclonal antibody. Encapsulating urea with a liposome allows the urea to incorporate into the cell more easily via lipid fusion. Furthermore, coupling with anti-VEGFR ensures that the urea selectively acts on VECs, to improve the efficiency of urea against hemangioma. Indeed, urea immunoliposomes significantly inhibited the proliferation of HVECs compared with urea or urea liposome (Table 1 and Figure 3).

The encapsulation rate is an important quality index of liposomes [11,12]. Liposome encapsulation is influenced by many factors, such as the drug’s solubility, lipid composition, the ratio of drug to lipid, and the ratio of oil to water when the liposome contains a water-soluble drug. The conditions were determined according to previous experiments to produce the urea liposomes: a phospholipid: cholesterol ratio of 4:1; a urea: lipid ratio of 1:60; and an oil: water ratio of 5:1. The urea liposomes showed a large unilamellar structure (Figure 1B), and the encapsulation percentage was 54.4%, which was slightly better than the figures reported by previous studies [11]. The coupling rate is an important index that reflects the quality of the immunoliposome. It is also influenced by many factors, including the coupling method, antibody type, and liposome quality [13]. The coupling rate of anti-VEGFR to the urea immunoliposome was 36.84%; it is expected that this can be further improved by optimizing the coupling conditions.

The etiology and development of hemangioma have not been elucidated [14]. Only some proteins and polypeptide growth factors have been reported to play important roles in the pathophysiology of hemangioma. Both VEGF and VEGFR are reportedly closely involved in the pathology of hemangioma. In our experiments, treatment with urea immunoliposome coupled with anti-VEGFR significantly inhibited the proliferation of HVECs: the inhibition rate was 99.91% on the 8th day and the doubling time could not be determined. Although treatment with urea liposome inhibited the proliferation of HVECs more than urea treatment due to the protection of urea by the liposome, its inhibition was less pronounced than that of the urea immunoliposome. We thought that in addition to targeting urea to HVECs, the anti-VEGFR antibody coupled to the urea immunoliposome could also block the effect of VEGF and induce death and atrophy in HVECs.

Recently, targeted therapy has been increasingly developed and applied to cancer therapy [15]. The inhibition of HVECs by urea immunoliposome has not been reported to date. The urea immunoliposome we developed has several advantages: urea encapsulated by liposome is more easily incorporated in HVECs; anti-VEGFR ensures the targeting of urea to HVECs in order to exert cytotoxicity on only these cells; and anti-VEGFR also blocks the effect of VEGF on HVECs to induce apoptosis and atrophy. The use of urea immunoliposomes resulted in the distinct and persistent inhibition of HVEC proliferation and could be used to treat hemangioma in the clinic.

## Conclusions

The urea immunoliposome we developed distinctly and persistently inhibited the proliferation of HVECs and can potentially be used in clinical hemangioma treatment.

## Abbreviations

DMEM: Dulbecco’s modified Eagle’s medium; DT: Doubling time; FBS: Fetal bovine serum; HVEC: Human vascular endothelial cell; VEC: Vascular endothelial cell; VEGF: Vascular endothelial growth factor; VEGFR: Vascular endothelial growth factor receptor; VIII-R Ag: Factor VIII related antigen.

## Competing interests

The authors declare that they have no competing interests.

## Authors’ contributions

JL and XD performed almost all the experiments and wrote the manuscript. ZW and GC supported the study by assisting with the design and interpretation of the work. Statistical analysis was performed by XD and XX. The overall supervision of the manuscript was completed by GC. All authors read and approved the final manuscript.
